# Methicillin Resistant Staphylococcus Aureus Shoulder Pyomyositis with Multifocal Lung Infiltrations

**DOI:** 10.3390/pediatric12030027

**Published:** 2020-11-17

**Authors:** Angela Troisi, Giulia Graziani, Alessandra Macaluso, Lorenzo Mambelli, Federico Marchetti

**Affiliations:** Department of Pediatrics, Santa Maria delle Croci Hospital, 48121 Ravenna, Italy; angela.troisi@auslromagna.it (A.T.); giulia.graziani@auslromagna.it (G.G.); ale_macaluso@libero.it (A.M.); lorenzo.mambelli@auslromagna.it (L.M.)

**Keywords:** pyomyositis, shoulder, MRSA, pneumonia

## Abstract

Pyomyositis is a rare condition in temperate climates. We present a case of *Methicillin Resistant Staphylococcus aureus* pyomyositis of the shoulder complicated by multifocal lung infiltrations, treated successfully with antibiotic therapy. After excluding shoulder septic arthritis, a low threshold of suspicion for the diagnosis of shoulder pyomyositis should be applied to patients with persistent fever, pain, and decreased range of shoulder motion. A prompt diagnosis and a rapid rise in antibiotic therapy are important to avoid local and systemic complications.

## 1. Introduction

Pyomyositis is an acute bacterial infection of the skeletal muscle that arises from hematogenous spread, and *Staphylococcus aureus* is the most likely causative organism.

We report the case of a 14-year-old girl with extensive muscular involvement and nodular infiltrates in both pulmonary fields with pleural effusion, secondary to an infection by *Methicillin Resistant S. aureus* (MRSA).

## 2. Case Report

A 14-year-old previously healthy girl was referred to our Emergency Department after three days of right shoulder pain. She presented with an altered state of consciousness, fever, dizziness, vomiting, and urticarial rashes. Clinical evaluation outlined the tenderness of the proximal part of the right arm with intense pain on the passive range of motion and decreased voluntary movements of the right shoulder. The patient did karate, but significant trauma was not reported; however, there was a slight bruise on the lateral surface of the right shoulder, which the girl reported subject to continuous microtraumatism secondary to martial arts (she used her right arm in the guard position when fighting).

Laboratory findings showed an increase in white blood cell count (leukocytes 15140/mmc with 80% of neutrophils), creatinine (1.35 mg/dL), C reactive protein (CRP) (59.5 mg/L, normal range < 5 mg/L), fibrinogen degradation products (3500 mcgr/L), and International Normalized Ratio (INR) (1.41). A shoulder X-ray was negative, while an ultrasound showed soft tissue swelling and edema of the shoulder girdle muscles.

Suspecting a localized infection of the right shoulder and an initial septicemic status, after collecting a blood sample for culture, broad-spectrum antibiotic therapy with ceftriaxone and clindamycin was started. 

Magnetic Resonance Imaging (MRI) confirmed extensive soft tissue and muscular signal abnormalities without bone and joint involvement (Figure S1). It showed nodular infiltrates in both pulmonary fields with pleural effusion, as confirmed by chest Computer Tomography (CT) (Figure S2). The patient developed symptoms of pneumonia a few days later, with coarse crackles on the left lung and bilateral reduction in hear transmission, particularly in the lower lobes. Tuberculosis infection and immunodeficiencies were ruled out.

Blood culture was found positive for a *Methicillin Resistant Staphylococcus aureus* (MRSA), resistant to beta lactams, cephemes, carbapenems, and clindamycin and susceptible to quinolones, glycopeptides, and oxazolidinones. Therefore, the antibiotic therapy was changed to vancomycin and ciprofloxacin. Linezolid was added eight days after admission for persistent fever.

The clinical conditions slowly improved, with complete recovery after ten days of overall treatment, as well as two days after starting Linezolid. Follow-up blood tests documented a progressive reduction in CRP. Thoracic X-ray and ultrasound documented the involution of nodular infiltrates and pleural effusion. After 20 days of parenteral therapy, oral antibiotic therapy with linezolid was continued for another 2 weeks.

A control MRI of the right upper limb performed after one month showed a reduction in edema in the right shoulder, with mild involvement of the muscle.

## 3. Discussion

Pyomyositis is a subacute, deep pyogenic infection of the skeletal muscle tissue, most commonly caused by *Staphylococcus aureus* species [1,2]. Originally described in tropical areas, it has been subsequently reported with an increasing frequency also in temperate climates [1,2,3].

Several predisposing conditions have been identified, including intense exercise, trauma, recent infection, diabetes, and immunocompromised state.

The pathogenesis of pyomyositis is not fully understood. Muscle trauma may lead to a muscle hematoma, which subsequently becomes colonized by bacteria during a transient bacteremia [4].

In our case, repetitive trauma due to karate may have caused the initial muscle cell damage with subsequent hematoma.

Bacteremia, sepsis, acute renal failure, Acute Respiratory Distress Syndrome (ARDS), and metastatic abscesses are the most frequent complications described. However, septic pulmonary emboli arising from primary deep tissue infections may be the cause of metastatic abscesses in the lungs [5].

Our patient presented a pyomyositis of the right upper limb caused by MRSA with septicemia. The onset of infection was characterized by local pain and systemic signs and symptoms (fever, dizziness, altered consciousness, elevated white blood cell count, CRP, creatinine, and elongation of INR) as an early stage of multiorgan failure, which resolved in 24–48 hours. The patient presented bilateral infiltrations and pleural effusion, probably due to hematogenous spread and septic embolization.

Over the last two decades, a massive spread of community-acquired MRSA (CA-MRSA) as the cause of pyomyositis has been documented in otherwise healthy children without risk factors [2].

Empirical antibiotic therapy should be started against principal bacteria according to the geographical area of reference. A specific treatment should be initiated once blood culture results are available. When we suspect an infection from CA-MRSA, the first line therapy is vancomycin, teicoplanin, or linezolid, especially against clindamycin-resistant CA-MRSA. Parenteral therapy should be continued until clinical improvement and the normalization of CRP. Thereafter, the treatment can be continued orally for at least two weeks. A surgical drainage is necessary if there is an abscess.

A radiological follow up through MRI is important in patients with complications, and huge extension of the infection [4].

## 4. Conclusions

Pyomyositis is a rare disease of the tropics showing increasing incidence also at our latitude. In our case pyomyositis was related to factors affecting the muscle itself, including strenuous exercise and continuous microtraumatism. The clinical course was complicated by multifocal lung infiltration. *Staphilococcus aureus* is the primary agent but also CA -MRSA and clindamycin resistance MRSA are on rise. Empirical therapy should be guided by prevalent resistance patterns. After excluding shoulder septic arthritis, a low threshold of suspicion for diagnosis of shoulder pyomyositis should be applied to patients with persistent fever, pain and decreased range of shoulder motion. A prompt diagnosis and a rapid rise of antibiotic therapy are important to avoid local and systemic complications.

## Supplementary Materials

The following are available online at https://www.mdpi.com/2036-7503/12/3/27/s1. Figure S1: right shoulder MRI showing STIR weighted signal intensity alteration of muscles with fluid collection and pulmonary infiltrates. Figure S2: hest CT scan showing bilateral alveolar infiltrates with ground glass aspect that vary from 7 mm to 2.5 cm consistent with septic pulmonary emboli.
