# Supplementary figures and images for: Methicillin Resistant Staphylococcus Aureus Shoulder Pyomyositis with Multifocal Lung Infiltrations
Source: Pediatr Rep. 2020 Nov 17;12(3):127–9. doi: 10.3390/pediatric12030027 (PMC7717662; doi:10.3390/pediatric12030027)

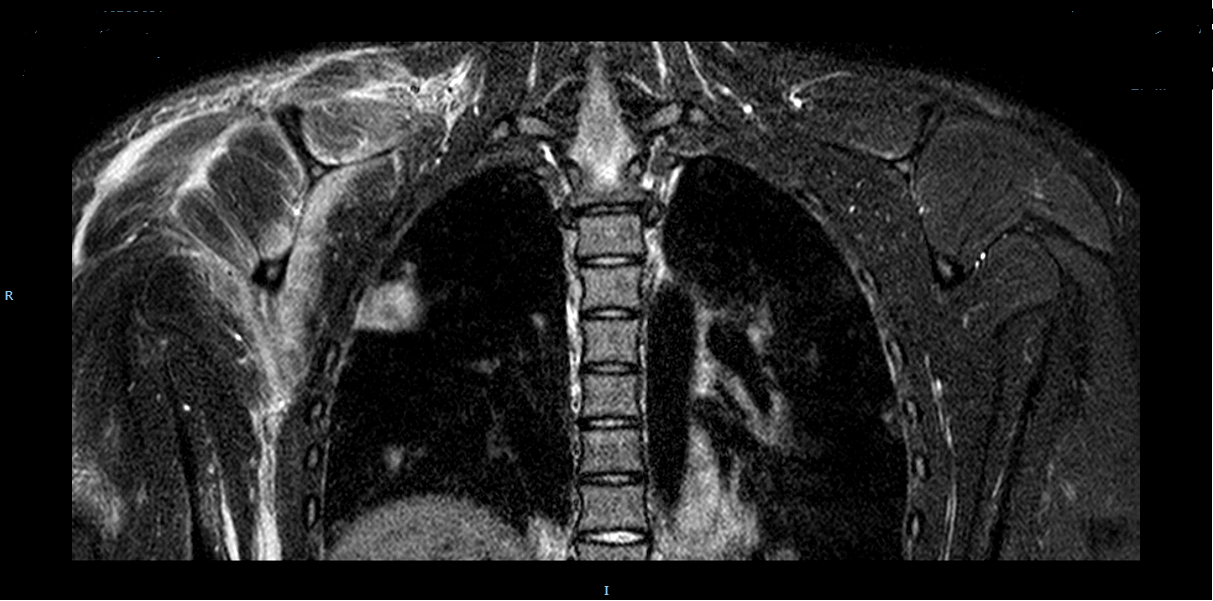

Supplement: Supplementary file 1 [file pediatrrep-12-00027-s001.zip › figure 1.v1.tiff]

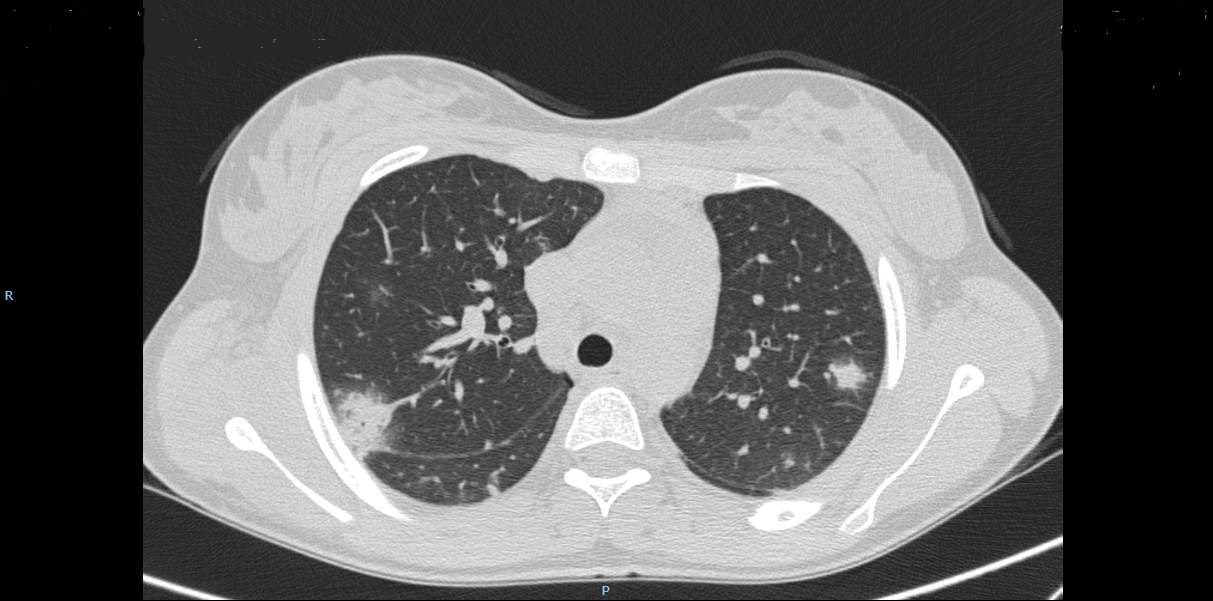

Supplement: Supplementary file 1 [file pediatrrep-12-00027-s001.zip › figure 2.v1.jpg]
